# Ninth International Medical Congress

**Published:** 1887-10

**Authors:** 


### AMERICAN JOURNAL

—OF—

### DENTAL SCIENCE.

VOL. XXI. THIRD SERIES—OCTOBER, 1887. No. 6.

ARTICLE I.

### NINTH INTERNATIONAL MEDICAL CONGRESS.

[Held in Washington, D. C., September 5, 6, 7, 8, 9 and 10, 1887.]

### MONDAY, SEPTEMBER 5TH—FIRST DAY.

The Congress assembled in Albaugh's Opera House, and was formally opened at 11 A. M., by His Excellency GROVER CLEVELAND, President of the United States, who said: "I feel that the country should be congratulated to-day upon the presence at our capital of so many of our own citizens, and those representing foreign countries who have distinguished themselves in the science of medicine, and are devoted to its further progress. My duty in this connection is a very pleasant and a very brief one. It is simply to declare that the Ninth International Medical Congress is now open for organization and for the transaction of business."

The address of welcome was delivered by Hon. Thomas F. Bayard, Secretary of State. In the name of his fellow-countrymen he expressed gratification at the visit of the delegates to Washington. The world is becoming acquainted and international intimacy is growing; a spirit of common brotherhood is increasing so that the word "stranger" will soon be obliterated from the vocabulary of civilization. If letters constitute a republic, science is a democracy. In the United States individual enterprise has produced great scientific institutions without the aid or interference of government. The proceedings of the Congress will be watched with interest by the sixty million people of this country.

Responses were made by the following gentlemen:

Dr. William H. Lloyd, of the Royal Navy; Dr. Leon Le Fort, of France; Professor Unna, of Germany; Professor Semmola, of Italy; Dr. Charles Reyher, of Russia.

Dr. Lewis A. Sayre, of New York, occupied the chair during the delivery of the address of the President of the Congress.

Dr. Davis began by paying an eloquent tribute to the memory of Austin Flint, M. D., LL. D., and continued as follows:

With a full consciousness of my own deficiencies, and still with a heart overflowing with gratitude, I thank you for the honor you have bestowed in selecting me to preside over the deliberations of this great and learned assembly. It is an honor that I appreciate as second to no other of a temporal nature because it has been bestowed, neither by conquest nor hereditary influence, nor yet by partisan strife, but by the free expression of your own choice.

The living human body, the chief object of your solicitude, not only combines in itself the greatest number of elementary substances and the most numerous organs and varied functions, so attuned to harmonious action as to illustrate the operation of every law of physics, every known force in nature, and every step in the development of living matter from the simple aggregation of protoplasm constituting the germinal cell to the full-grown man, but it is placed in appreciable and important relations with the material objects and immaterial forces existing in the world in which he lives.

Hence a complete study of the living man, in health and disease, involves a thorough study, not only of his structure and functions, but more or less of every element and force entering into the earth, the air, and the water, with which he stands in constant relation.

The medical science of to-day, therefore, embraces not only a knowledge of the living man, but also of such facts, principles, and materials gathered from every other department of human knowledge as may increase your resources for preventing or alleviating his suffering and of prolonging his life.

The time has been when medical studies embraced little less than the fancial theories and arbitrary dogmas of a few leading minds, each of which became for the time the founder of a sect or so-called school of medicine, with his disciples more or less numerous. But with the development of general and analytical chemistry, of the several departments of natural science, of a more practical knowledge of physics, and the adoption of inductive processes of reasoning, the age of theoretical dogmas and of medical sects blindly following some more plausible leader passed away, leaving but an *infinitesimal* shadow yet visible on the medical horizon.

The address closed with an appeal for the collective investigation of the phenomena of disease.

SECTION ON DENTAL AND ORAL SURGERY.

Jonathan Taft, M. D., of Cincinnati, O., President.

*Secretaries*—A. M. Dudley, M. D., of Salem, Mass.; F. H. Rehwinkel, M. D., of Chillicothe, O.

MONDAY, SEPTEMBER 5TH—FIRST DAY—AFTERNOON SESSION.

The President welcomed those present.

Drs. I. V. Metnitz, of Austria; B. McLeod, of Scotland; and Greverts, of Holland, replied in behalf of the countries they represent.

The President then delivered his address, in which he reviewed the progress of dentistry in the last fifty years, and concluded by saying that although the past record was an excellent one, yet the goal is not yet reached. He urged the profession, through those present, to work in all earnest for a yet higher standard.

Dr. R. J. Porre, of Cincinnati, O., read a paper on "chronic pyæmia from dental origin."

The history of the case is as follows: The patient, male, good constitution and habits, suffered for the last thirty years from neuralgia, besides having constantly recurring furuncles and eruptions in various parts of the body, which would often for months become running abscesses. He experienced burning and itching eruptions of hands and feet, which would finally change to stubborn ulcerations. His bowels were either stubbornly constipated or exhaustingly loose. He suffered from frequent rigors and febrile attacks of varying intensity, profuse night-sweats, retention of urine, serious constriction of the bowels and urethra. Lancinating pains darted from the maxilla of right side to bowels, bladder, limbs, hands and feet, or to whatever part that was locally affected at the time. This latter peculiarity, together with the discovery of a little pus exuding from the locality of the wisdom tooth, led to a final correct diagnosis of his case.

The tooth referred to was extracted, and a speedy and complete recovery followed. As other sources leading to pyæmia and having their starting-point in the oral cavity may be mentioned pyorrhæa alveolaris, alveolar abscess, abscess of the antrum, and dental caries.

The doctor related ten other cases similar to the above, which all yielded to the simple remedy of removing the offending tooth.

Dr. J. Frank Lydston, of Chicago, Ill., said that both physicians and dentists should appreciate the important relation which morbid conditions of the mouth and jaws, and especially those which may be produced by septic absorption, bear to different general conditions. Septic matter is quite generally found about the roots of teeth, and may, under favoring circumstances, be absorbed into the blood, and there produce disturbances of greater or less degree.

The paper was further discussed by Drs. Walker, of London, England; Barrett, of Buffalo, N. Y.; W. J. Younger, of San Francisco, Cal., and Chance, of Oregon.

---

TUESDAY, SEPTEMBER 6TH—SECOND DAY—MORNING SESSION.

Dr. William Carr, New York, N. Y., gave a *clinic* on the "treatment of fractures of the maxillæ with modified interdental splint."

The majority of fractures of the inferior maxilla occur in the body rarely at the symphysis menti, but usually directly anterior or posterior to the mental foramen. A noticeable fact in connection with these fractures is that the victim rarely applies for treatment for several days succeeding the injury. He realizes that some of his teeth are loosened and also that he is painfully bruised, but does not seek surgical aid until he becomes alarmed by the increased inflammatory condition of the parts. There is but little difficulty in establishing a correct diagnosis, as usually the following symptoms are present—great pain in the effort to open and close the mouth, swelling, crepites, inflammation, inability to masticate, and marked irregularity of the teeth.

*Treatment.*—It is identical with that of other fractures, namely, to bring the parts into apposition and retain them firmly until ossification is completed. For treatment of fractures of the maxillæ there is nothing superior to the interdental splint. When properly adjusted, speedy union may be secured without deformity of the jaw or irregularity of the teeth. Before taking the impression a careful examination of the parts should be made. Loose teeth and spicula of bone should be removed, and the parts should then be brought as nearly as possible to their normal position. An accurate impression should be made with impression-compound or wax. The material used should be as warm as the patient can bear it, in order to prevent unnecessary pain and also to prevent further displacement of the parts. The splint is made of vulcanite and covers all the teeth of the lower jaw, and all the teeth posterior to the canine in the upper jaw—leaving a space of about three or four lines through which the patient may receive nourishment. Small holes are drilled in the splint over the grinding surface of each molar for the purpose of ascertaining whether its adjustment is proper.

The splint should first be adjusted to the sound jaw, then gently bring the fractured jaw into position until it has passed about two-thirds of the length of the teeth—then with a quick, firm motion bring the parts into position. Next apply a four-tail bandage, which should be retained from three to five days; after this time, in the majority of cases, it may with safely be removed during the day but should be replaced at night until the removal of the splint. The patient should be furnished with an ordinary rubber syringe, and instructed to keep the mouth thoroughly cleansed. For disinfectants I use peroxide of hydrogen three per cent. solution, or a solution of bisulphate of soda in the proportion of ʒ j to ℥ j, of water.

In ordinary cases the splint should be retained for three or four weeks, according to the physical condition of the patient—unless unforeseen complications should arise. The application of the splint, combined with thorough cleanliness, will usually be all the treatment required.

The advantages, besides those previously stated, are that the patient experiences but little pain and inconvenience and can, as a rule, attend to his business almost immediately after the splint is applied.

It is not necessary that all the teeth, nor, indeed, that any should be present in the mouth in order to make this splint serve its purpose. In the first case the rubber can be made to take the place of the missing teeth, and in the latter case a perfect adaptation of the splint to the alveolar ridges can be secured, and will be found to keep the parts in perfect apposition.

Should it be deemed advisable to place a splint in position within an hour or two after seeing the case, one can be constructed entirely of ordinary gutta-percha, with just enough wire inside to stiffen it. Dr. Carr demonstrated this last method—it is very simple and can be made by any surgeon.

A number of gentlemen examined the principle and pronounced it very satisfactory in every way, the main points being its simplicity of construction, its effectiveness, and the ease with which it is adjusted and worn by the patient.

Dr. E. Brasseur, of Paris, France, read a paper on "the use of air in dental therapeutics."

The reader urged that the ordinary means, such as bichloride and biniodide of mercury and carbolic-acid crystals, for destroying microbes in the oral cavity and, especially, in carious cavities of teeth, should be supplemented by the use of hot air.

Dr. C. A. Brackett, of Newport, R. I., discussed the paper at some length, laying considerable stress on the efficacy of crystallized carbolic acid as a germicide in carious cavities in teeth.

Other discussions followed, by Drs. James Truman and W. H. Morgan.

AFTERNOON SESSION.

Dr. Junius E. Cravens, of Indianapolis, Ind., read a paper on "the management of pulpless teeth."

This system is based on the proposition that a pulpless tooth is not necessarily dead. The pulp being devitalized, the tooth still retains life through its pericementum. The usual course of treating pulpless teeth with escarotics and irritants cause irritation and final destruction of the pericementum, and the result is that the tooth, instead of being preserved, will act as a foreign body, and will be thrown off by nature through abscesses; or, worse still, will lead to no end of nervous derangements.

The treatment suggested by the reader is to thoroughly cleanse the pulp canal, and at once hermetically seal it with tin-foil.

The paper was discussed by Dr. Thomas Fillebrown, of Portland, Me. He did not agree with the esayist in the method outlined in the paper. The doctor gave a short synopsis of the method he employs in treating pulpless teeth, which, by the manner in which it was received by the Section, seemed to be the one generally pursued.

Dr. A. W. Harlan, of Chicago, Ill., followed, and likewise objected to the views expressed by the essayist. A dead pulp produces no irritation in the canal; the disease which it causes is beyond. If you could mechanically displace an odor—which the speaker denied—and should then fill the root-canal without any disinfection, disaster would inevitably follow unless there should be a fistulous outlet.

Dr. W. C. Barrett, of Buffalo, N. Y., in discussing the paper, stated whether viewed from the standpoint of pathology or etymology the paper is alike remarkable. That such a mass of absurdities could be presented at a meeting of the world's representatives in dentistry is to me astounding, and I protest against its acceptance as the standard by which to judge the intelligence of American dentists. Why the exploded dogmas of twenty-five years since should be gravely and in all sincerity presented at such a meeting as this is, I must confess, something for which I was not prepared. The assertion that a closed chamber in which exists the septic debris and the products of decomposition of a tooth-pulp should not be opened and evacuated, I can scarcely believe is made in calm earnest. The essayist has exhibited his complete ignorance of the progress of the past century.

Modern antiseptic pathology has taught us certain facts, and among these is the knowledge that the first step in the treatment of aseptic cavities is complete drainage; second, disinfection and the removal of all the products of disorganization; third, destruction of septic organisms; and finally, the complete sealing of the cavity against further infection. These comprise the essential steps in the treatment of septic root-canals. I will not insult the intelligence of those present by presuming to enlarge upon this and by going into the details of treatment, for this is not a body of tiros. But I do object to a consideration of the subject from the low standpoint of this extraordinary paper.

Dr. T. E. Weeks, of Minneapolis, Minn., read a paper on "matrices as adjuncts in filling teeth."

The essayist reviewed the different appliances for simplifying what would otherwise be very laborious operations. A perfect matrix should be simple in construction, cheap, easily adapted, and not too stiff, so that when applied it will yield just enough to allow sufficient gold to pass beyond the walls of the cavity for a good finish.

Dr. F. H. Guilford, of Philadelphia, Pa., in a few brief remarks, indorsed the sentiment expressed in the paper.

---

### THIRD DAY, WEDNESDAY, SEPTEMBER 7TH—MORNING SESSION.

Dr. Pradere, of Lyons, France, read a paper on "phthisis cured by the continuous application of medicine to the palate."

Immediately after the paper was read, Dr. James Trueman, of Philadelphia, Pa., moved that it should *not be accepted by the Section*, but should be referred, without discussion, to Section I., in General Medicine; also embodying that the Executive Committee be censured for allowing such a paper to come before the Section. The motion was seconded by Frank Abbott, M. D., of New York, but the Chair ruled that, inasmuch as the Executive Committee had seen fit to admit this paper, it would be out of order to put the motion to the house. Dr. Trueman dissented from the decision of the Chair and renewed his motion. The question being then called, it was voted to refer the paper to Section I.

CLINIC.

A number of gentlemen gave clinics in the treatment of diseased conditions of the oral cavities, and others demonstrated their methods of filling teeth and constructing artificial dentures for patients. These clinics are spoken of as the most successful features in this Section, and it is but just to say that a good deal of credit is due to Dr. C. F.W. Boedecker, of New York, for the result.

Dr. Metnitz, of Vienna, Austria, read a paper on "osteomyelitis."

The main feature of the paper was the report of two cases from practice.

The history of the first case was as follows: In October, 1886, a lady, aged forty-three, had two teeth extracted. A few days later she suffered with chills, which were followed by slight mental disturbances. The seventh day the patient became unconscious, in which condition she was brought to the hospital. Examination revealed that there was a large swelling over the left cheek, extending to the temporal region; the skin covering this swelling was tense and pale in color; thesclerotic was highly colored (yellow,) and the skin showed yellow tinge; the pupils were without reaction. The odor of the breath gave evidence of necrosis. The submaxillary glands were very much enlarged, and the neighboring tissues infiltrated. There was unconscious urination and defecation. Death occurred the following day. The postmortem cxamination showed the membranes of the brain to be thickened and traversed by numerous vessels. The left hemisphere was covered by a layer of pus, and the right hemisphere showed considerable pus along the track of the vessels as well as several pus-depots. The brain-substance was quite soft. The examination of the oral cavity disclosed that of the two teeth extracted the upper alveolus had almost entirely filled up with healthy granulations, whereas the lower was filled with pus. The mucous membrane in the region of this diseased alveolus was very much discolored and could be easily removed in pieces. The probe discovered nothing but dead bone. All the muscles of the neck which are attached to the left side of the lower jaw were infiltrated with pus. The periosteum was separated from the left side of body and ramus of the jaw. The alveolus of the extracted wisdom-tooth communicated by two good-sized openings with the marrow-cavity, and the marrow itself was discolored and infiltrated with fat. The cause of this extensive destructive action is no doubt to be looked for in the unclean condition of the alveolus after the extraction. Sections of the jaw show that the medullary canal was very much enlarged.

Rocher, Rosenbach and Busch, in experimenting on animals, have found that it is impossible to produce an acute pus-forming osteomyelitis either through traumatic injury or chemical and mechanical irritation, but that such a condition can readily be brought about by infecting the fresh wound in the bone by any decaying substance.

The second case was one of multiple osteomyelitis. The patient, male, aged seventeen, suffered from an attack of osteomyelitis of the humerus, the ulna, and the lower jaw. According to Billroth, it is not settled whether this condition (multiple osteomyelitis) is due to septic influences acting on various places at the same time, or whether the infection dates from one point.

Death in this case, as in the first, was directly due to acute suppurative meningitis. When we have to deal with a simple inflammation, energetic antiseptic treatment will prove quite sufficient. In severer cases of osteomyelitis Billroth advises that the seat of disease be reached as soon as possible —the pus evacuated, the cavity thoroughly disinfected, and dressed with antiseptic dressing. Many cases present no actual depots of pus, or abscesses, but simply an infiltration of the narrow. In such cases Billroth holds it of little value to open into the medullary canal Neither does he advocate disarticulation or resection, because, in the first place, the exact extent of the disease cannot be foretold, and, secondly, the medullary substance of a patient suffering from osteomyelitis is in such a susceptible condition that a new injury would almost certainly prove fatal.

This paper was read by the essayist in German. No discussion followed.

Dr. Jenison, of Minneapolis, Minn., read a paper on "art in dentistry."

The essayist advocated the restoration in gold of all teeth that had been destroyed by caries, thereby improving both their usefulness and beauty.

In constructing artificial dentures some time should be given to the restoration of the features of the patient, and for that purpose single and not section teeth should be used.

Dr. John Allen, of New York, discussed the paper, taking up the main points to be observed in constructing an artificial denture. He closed his remarks by saying that inasmuch as the countenance reveals the thoughts of a person, great care should be exercised in restoring lost features.

THIRD DAY—AFTERNOON SESSION.

Dr. R. R. Andrews, of Cambridge, Mass., read a paper on "the origin of the dental fibril, illustrated by aid of stereopticon.

Dr. Andrews described his process of preparing and mounting the specimens for the microscope, which differed in no essential respect from the latest methods employed by others for that purpose.

In speaking of the formation of the fibrils, the essayist says there are two kinds of odontoblasts—those which are square toward the dentine, and others, just by the sides of the first mentioned, which are pear-shaped. From these latter, and not from the first (or square end ones), originate the dental fibril.

The stereopticon views presented by the doctor showed very clearly with what patience, earnestness and intelligence the essayist worked to establish his view of the question. And the hearty appreciation accorded him by the Section was well merited.

Dr. Frank Abbott, of Mew York, in opening the discussion, paid a high tribute to the reader of the paper for the hard work done in behalf of his specialty. In order to understand the process by which the dental fibril is produced, it is necessary for us to consider the matter from the third to the fifth month of intra uterine life, at which period of the existence of the fœtus the papilla of teeth are so far developed that a material change is observed to be taking place. The papilla is a mass of myxometous tissue, liberally supplied with medullary elements. In some instances at three months, at others as late as the fifth of intra-uterine life, a coalescing of several of the medullary corpuscles into one may be observed upon the periphery of the papilla adjacent to the enamel organ, which at this period may be observed forming a cap upon the papilla. The united medullary corpuscles are known as odontoblasts. The impression has generally prevailed among histologists and embryologists, that the odontoblasts were directly formed into dentine. This theory, through recent researches, has been proven to be incorrect. The odontoblasts, when viewed with a power of 1,200, show a delicate reticulum, which unites the nuclei with the walls of each corpuscle and with each other. The reticulum, as well as the walls of the odontoblasts, are the living matter which remains as the living portion of the dentine. Before the beginning of the deposition of lime salts, the odontoblasts are re-converted into medullary substance. As such they receive the calcareous basis-substance, and thus a certain territory of the papilla becomes dentine. While this process of calcification is going on, another row of odontoblasts makes its appearance, from the sides and ends of which prolongations of the living matter may be seen running into the canaliculi of the dentine already formed. A spindle or pear shaped odontoblast gives off one, while those with broad ends give off two, three, and even five, prolongations. If the views advanced in the paper were correct, it would necessarily follow that territories of considerable size would be left in the dentine with no canaliculi whatever; nor is there any provision for furnishing these territories with any living tissue.

Dr. Fletcher, of Cincinnati, O., read a paper on "protective dentine; illustrated by stereopticon."

This paper was listened to with great interest by the Section. The slides which were shown on the screen showed the different kinds of protective dentine, and the essayist gave his views of how these different efforts on the part of nature to protect herself are brought about.

Dr. W. X. Sututh, of Philadelphia, agreed with the essayist in the practical conclusion drawn· he supplemented the reader's remarks by stating that the odontoblasts remain after the development of the dentine, but can be stimulated to produce or perform their function of forming protective dentine.

Dr. W. H. Atkinson, of New York, complimented the gentlemen on producing such well-digested papers.

Dr. J. Howard Mummery, of London, England, exhibited "photo-micrographs of all the structures of the tooth," and explained the best method of producing them.

---

THURSDAY, SEPTEMBER 8TH—FOURTH DAY—MORNING SESSION.

MICROSCOPY.

Professor Frank Abbott, of New York, and Dr. R. R. Andrews, of Cambridge, Mass., were in charge of this department. Every facility was afforded the members of this Section to acquaint themselves with dental microscopy, both physiological and pathological. Among the ground specimens shown by Professor Abbott were those of carious teeth, congenital pathological enamel, hyperostosis (osteomas) of the roots of teeth, and deposits of secondary dentine. Dr. Andrews exhibited serial slides of the developing teeth, and the development of the dental fibril. About forty negatives from his photomicrograph were especially interesting and valuable.

CLINICS.

About thirty gentlemen gave clinics in filling teeth with gold, pivoting teeth, constructing artificial dentures, and treating (surgically) diseased conditions of the gums. It would require too much space to enumerate all the gentlemen who acquitted themselves in such a creditable manner.

C. L. Goddard, A. M., D. D. S., of San Francisco, Cal., read a paper entitled, "pain in the temporo-maxillary joint caused by irregularity of the teeth.

Patient, thirty years old, experienced pain in temporo-maxillary joint during mastication, which was caused by straining the muscles and ligaments, owing to masticating with the jaw protruded. When the teeth were brought together, as in the act of eating, the incisors alone touched, and the bicuspids and molars were about one-eighth of an inch apart. The treatment employed consisted in spreading the upper teeth and thereby securing a proper articulation.

Dr, E. S. Chisholm, of Tuscaloosa, Ala., read a paper entitled "the influence of weather changes on the human organism."

After carefully noting the influence exerted by temperature, humidity, and electricity, the author concludes that by far the greatest power over human organism is exerted by atmosphere pressure. In support of this theory he submits two arguments. The normal atmospheric weight on man is 14.7 pounds to the square inch at the sea level. The body is sustained by an equal power of resistance, wisely provided. If the pressure be less, the surface of the body will be distended, and the superficial circulation less restrained. This change can be brought about by exposure to great altitude, as well as by natural physical causes, when the circulation will be disturbed just the same. Any undue pressure on a portion of the body may then be felt. May not this disturbance of tension on soft tissues which are fixed to the bony framework of man, or where disease has a seat in periostinal and ligamentous attachments, be liable to greater inflammation? Or when a nerve of a tooth, which in a state of health is inclosed in a bony chamber (which has no expansive liberties, nor needs them as long as health continues), becomes exposed through a small aperture; when the normal atmospheric balance is lowered, the nerve has a tendency to be drawn through the aperture and takes on inflammation, probably followed by congestion and complete devitalization.

A report from the Pennsylvania Hospital, some years ago, on the observation of barometric pressure in surgical operations, shows that in 259 operations the barometer was ascending in 102, descending in 123, and standing in 34. Fifty-four of the whole number were fatal, 11 having been operated on with barometer ascending, 25 when descending, and 8 when standing.

AFTERNOON SESSION.

The Section was honored by a visit from the President of the Congress, Dr. N. S. Davis. In introducing him, President J. Taft recounted the efforts that had been put forth by Dr. Davis to secure recognition to the Dental Section. To him more than to any other man in the medical profession is due the credit for having removed the obstruction in the way of the dental profession.

Dr. Davis replied: Twenty-two years ago I had the pleasure of entertaining the members of the American Dental Association, then assembled at Chicago, at my house. On that occasion I expressed the hope that some day, in the near future, we might meet on equal grounds. My hopes of that day are realized to day. At the last meeting of the American Medical Association, when the question was brought up to admit dentists holding their degree from a recognized institution, it met with no opposition. The action of that body has forever removed the obstacle which had been in your way, and you are now on an equal footing with your medical brethren. He congratulated the members for the interest they took in the advancement of the healing art, and closed by warning them not to fall into ' schools," but to meet everyone on the broad field of science.

Dr. E. S. Talbot, of Chicago, Ill., read a paper on "etiology of irregularities of the jaws and teeth."

This paper was very exhaustive and thoroughly well prepared. The writer showed throughout an intimate acquaintance with the subject, and the Section was not iacking in appreciation of his efforts.

The paper was discussed by Dr. W. C. Barrett, of Buffalo, N. Y.

A paper by Dr. J. J. R. Patrick, Belleville, Ill., on "Irregularities," was read by title; also one by Dr. E. H. Angle, Minneapolis, Minn., entitled "Notes on Othodontia, with a New System of Regulation and Retention;" also by Dr. L. C.

Ingersoll, Keokuk, Ia., on "Inflammation of the Oral Tissues."

---

FRIDAY, SEPTEMBER 9TH—FIFTH DAY—MORNING SESSION.

CLINICS.

A very interesting clinic was given by Dr. A. R. Starr, of New York, in capping the exposed pulp of a tooth.

It is only of late years that any progress has been made in so treating and protecting an exposed tooth pulp so as to preserve its vitality. The operation caused the patient very little pain, and all those who have had the opportunity to see it agreed that it was a success.

Dr. William J. Younger, of San Francisco, implanted a tooth. Great diversity of opinion was expressed as to the ultimate result of this method of thus supplying the lost teeth.

Dr. R. B. Adair, of Gainesville, Ga., completed the treatment of a case of pyorrhœa alveolaris.

Thirty two other gentlemen gave clinics on filling teeth and constructing artificial dentures.

Prof. F. Busch, of Berlin, Germany, read a paper on "the comparative pathology of the teeth, with special reference to the tusk of the elephant."

A number of specimens were shown by the doctor to illustrate his paper. At the close of his paper the doctor exhibited an instrument for removing the small birth-marks so often found on the face. The instrument have a circular cutting edge ranging in size from one-fourth to half an inch in diameter, and fit into the dental engine, The mode of operation consists in selecting a knife the size of the mark to be removed, and placing it upon it, quickly revolving it. It will take only a moment to accomplish this, and is said to be almost painless. The only dressing the doctor applies is dry cotton, which he leaves in position for from six to eight days, at the end of which time the part is perfectly healed.

Discussion of the paper was taken part in by Drs. Wm. H. Atkinson, of New York, and W. C. Barrett, of Buffalo, N. Y.

Dr. E. Andrieu, Paris, France, read a paper on "the sixth year molar."

The essayist held that the sixth year molar being in development, eruption, and structure an organ of transmission from the temporary to the permanent set, it should be extracted when the permanent teeth are in position. More room is gained by this procedure, and a longer period of usefulness insured to those remaining,

Dr. L. D. Sheperd, of Boston, Mass. The doctor expressed his regret that such sentiments should still be prevailing among the profession. He cited cases from practice, when the extraction of these teeth caused not alone loss of valuable masticating surface, but actual irregularity of the ones remaining.

Dr. Paul Dubois, of Paris, France, stated that he wished the Section to note that the ideas expressed in the paper were not carried out by the dentists in France.

Professor Frank Abbott, of New York, could see no difference in structure between the sixth-year molar and the other permanent teeth under the microscopie, but a very marked difference was apparent between that and the temporary teeth.

A paper by Dr. Th. David, of Paris, France, on "Aphthous Stomatitis," was read by title.

Dr, J. S. Marshall, of Chicago, Ill., read a paper on "operation for the cure of a persistent neuralgia of both temporo-maxillary articulations, and reflected pain in the right brachial plexus.

History.—Patient, female, forty-two years of age, was operated on some eight years before for the removal of an osteo-sarcoma of the right inferior maxilla. Extensive suppuration followed, and the wound did not heal for several months. Considerable cicatricial tissue was formed, and the jaw displaced to a considerable extent. Neuralgia dated from the healing of the wound, and the conclusion arrived at was that the irregular position of the jaw was the cause. An operation for the relief of this condition was performed, and the neuralgia ceased. The doctor described the steps of the operation at great length, but failed to show that it differed from methods generally employed.

The following papers were read by title: "Articulation of Artificial Teeth," by Dr. H. L. Cruttenden, of Northfield, Minn.; "Power in Dentistry," by W. St. George Elliot, M. D., of London, England; "Porcelain Filling," by Dr. E. C. Moore, of Detroit, Mich.

Dr. Alton H. Thompson, of Topeka, Kan., read a paper entitled "does function control the evolution of structure?"

Some time since, Dr. C. N. Pierce discussed a subject similar to that at the head of this paper. After noticing the mechanical forces involved in and influencing the evolution of the teeth, he says: "Those cumulative forces are utilized through heredity, and while so potent in tooth-evolution, exert a similar influence in the development and modification of all other structures and organs. All departments of biology recognize the fact that heredity, adaptation, and growth, being of special importance in the evolution of the organic body, must therefore be regarded as especially formative functions. Adaptation to environment might be called the ancestor of function, as function is of organization. Illustrations of modifications of structure in response to function are very numerous in which it is shown how an organ may be completely changed or a mere rudiment be fully developed by the demand for the performance of a function unknown before."

A century ago Lamarck laid down the following laws concerning the development of organic life, which we, with all our accumulated facts, can change but little. In his second law he said that the production of a new organ in an animal body results from the supervention of a new want continuing to make itself felt, and a new movement which this want gives birth to and encourages. Third law: The development of organs and their force of action are constantly in ratio to the employment of those organs. Fourth law: All that has been acquired, laid down, or changed in the organization of the individual, in the course of this life, is commenced by generation and transmitted to new individuals which proceed from those which have undergone those changes." Altered wants lead to altered habits which result in the formation of new organs, as well as in modification and growth of those previously existing.

In our study of the subject, we will confine our observations to that field in which we, as dental and oral specialists, are most interested the teeth.

Everything is made for a purpose, and the purpose must precede the thing made for effecting the purpose. Nature plans her work as deliberately as man plans his actions. The means for accomplishing an end are not the cause of execution, but the effect. The organ is the effect, and function is the cause of structure. As food-selection is the cause of the function, so the function of the acquisition and preparation of food is thus the cause of the masticating apparatus.

If function is the cause and support of structures, if an organ develops or atrophies as it is used or disused, if the impulse of active employment dictates the evolution of parts and tissues in succeeding generations, if organs have been suppressed through disuse or remain in various forms as mere rudiments—their function having passed away—then, indeed, must the teeth of man be tending toward final and inevitable suppression.

If in the future of physical education there shall be found a place for education in the accomplishment of masticating food, then, indeed, may the teeth be improved and developed by exercise, as the muscles and lungs are improved and developed.

Owing to the time for adjournment having arrived, no discussion was had on this paper.

The President congratulated the members on the interest they had shown throughout the session, and hoped great good might come from this Congress, both to the profession and their patients.

A vote of thanks was voted to President L. S. Davis for the exertion he put forth in behalf of the dental section.

Also a vote of thanks was offered to President J. Taft and the Executive Committee.

[From advance slips supplied by *The Medical Record*, of New York, from its special report.]

